# Paediatric Rotary Files and Dentinal Crack Formation in Primary Teeth: A Systematic Review

**DOI:** 10.7759/cureus.77033

**Published:** 2025-01-06

**Authors:** Syed Sulaiman, Victor Samuel A, Kavitha Ramar, Sujitha Ponraj, Arya Varghese

**Affiliations:** 1 Pediatric Dentistry, Sri Ramaswamy Memorial (SRM) Kattankulathur Dental College and Hospital, SRM Institute of Science and Technology, Chennai, IND; 2 Pedodontics and Preventive Dentistry, Sri Ramaswamy Memorial (SRM) Kattankulathur Dental College and Hospital, SRM Institute of Science and Technology, Chennai, IND; 3 Pediatric and Preventive Dentistry, Sri Ramaswamy Memorial (SRM) Kattankulathur Dental College and Hospital, SRM Institute of Science and Technology, Chennai, IND

**Keywords:** dentinal microcracks, micro ct in endodontics, niti files, paediatric rotary files, primary teeth root canal

## Abstract

Nickel-titanium (NiTi) rotary files are widely used in endodontics due to their flexibility, durability, and efficiency in shaping root canals. However, concerns have been raised regarding their potential to cause dentinal cracks, particularly in primary teeth with their unique structural vulnerabilities. This systematic review aims to evaluate the prevalence, characteristics, and implications of dentinal cracks associated with the use of NiTi rotary files in primary teeth. A comprehensive literature search across multiple databases yielded 5,147 articles, with five studies ultimately meeting the inclusion criteria. These studies primarily investigated various NiTi file systems, such as ProTaper, WaveOne Gold, Reciproc, and Kedo-S, examining their impact on crack formation. Findings indicated that systems with larger tapers and aggressive cutting actions, such as ProTaper, exhibited a higher incidence of dentinal cracks compared to paediatric-specific or more conservative designs like Kedo-S. The clinical implications of these findings underscore the importance of selecting appropriate instrumentation to minimise structural damage in primary teeth. The review advocates for standardised methodologies in future studies to enhance comparability and calls for innovations in paediatric-specific rotary file systems to improve treatment outcomes.

## Introduction and background

Nickel-titanium (NiTi) rotary files have transformed endodontic instrumentation by providing significant advantages over traditional stainless-steel files [1]. Renowned for their superior flexibility, durability, and fracture resistance, NiTi rotary files enable more efficient and precise root canal shaping [2]. Their availability in various shapes, sizes, and tapers allows them to adapt to the complex anatomical challenges of root canal systems, ensuring effective cleaning and shaping even in intricate or curved canals [3].

The use of these instruments in paediatric dentistry comes with certain risks, particularly the potential for dentinal crack formation. This complication often goes unnoticed but can undermine the structural integrity of primary teeth [4]. Such cracks may develop inadvertently during root canal preparation, especially when mechanical stresses exceed the dentin's natural resistance. Recognising the causes, characteristics, and clinical significance of these cracks is essential to reducing the likelihood of adverse outcomes in paediatric patients [5].

Primary teeth, with their distinct anatomical and developmental characteristics, warrant special consideration during endodontic treatment [6]. The thinner dentinal walls and more complex root structures of primary teeth increase their susceptibility to cracks during instrumentation. Despite the widespread use of NiTi rotary files in paediatric dentistry, limited research has been dedicated to understanding the specific risks associated with dentinal crack formation in primary teeth. Closing this research gap is essential for refining treatment strategies that safeguard the long-term health of paediatric patients.

This systematic review helps to bridge the knowledge gap by examining the existing literature on dentinal crack formation caused by NiTi rotary file instrumentation in primary teeth. By synthesising current evidence, it seeks to evaluate the prevalence and characteristics of these cracks and offer insights to guide clinical practice. The findings are intended to help refine treatment protocols and improve outcomes in paediatric endodontics.

This review lays the groundwork for future research by identifying key areas for further exploration. The findings can inform clinical trials that evaluate the real-world outcomes of NiTi file use in primary teeth, including their long-term effects on tooth survival and crack progression. Potential directions for advancement include the development of paediatric-specific rotary files with improved designs tailored to the unique anatomical features of primary teeth, such as shorter lengths and smaller tapers. Additionally, standardising protocols for assessing and reporting dentinal cracks could enhance consistency and comparability across studies.

Investigating preventive strategies and innovative technologies, such as real-time feedback systems to minimise mechanical stress during endodontic procedures, represents another promising avenue for future development. Hence, this systematic review aims to enhance understanding of the factors contributing to dentinal crack formation in primary teeth.

## Review

Research question

Centred on the "PICOS" procedure, research that examined the occurrence of dentinal microcracks consequent from endodontic files manufactured with varied NiTi alloy composition and cross-sectional geometry was included.

The study focuses on human primary teeth, specifically investigating the impact of using rotary NiTi for instrumentation. The intervention is compared against various other file types, including heat-treated files, titanium-treated files, gold heat-treated files, nickel-titanium heat-treated files, and EDM-treated files. The primary outcome of interest is the propagation of cracks in the root dentin of these teeth. This includes any form of dentinal defects, such as dentinal cracks, craze lines, microcracks, incomplete cracks, complete cracks, or fracture lines along the surface of the root dentin.

Search strategy

The systematic review began with an extensive literature search across multiple electronic databases, including PubMed, Google Scholar, LiLac, Scopus, and EBSCO. In addition to these sources, manual searches were conducted to identify studies examining crack formation in root dentin of human primary teeth caused by rotary nickel-titanium instruments. The search strategy employed various keyword combinations and Medical Subject Headings (MeSH) terms related to microcracks, NiTi files, and dentin. This comprehensive search initially yielded 5,147 articles. After removing duplicates and excluding studies published in languages other than English, 5,145 unique records were retained for screening.

Eligibility criteria

The selection criteria for this research were carefully designed to ensure relevance and accuracy. Only in vitro studies conducted on extracted human primary teeth were considered for inclusion. These studies had to involve root canal preparation using nickel-titanium (NiTi) rotary files, as the focus was on assessing their impact on dentinal crack formation. Crack evaluation methods in the included studies were required to use precise techniques, such as dye penetration, digital microscopy, advanced microscopic imaging, stereomicroscopy, scanning electron microscopy (SEM), or micro-computed tomography (micro-CT). Additionally, only articles published in English were included in the analysis.

Conversely, strict exclusion criteria were applied to maintain the study’s focus. Studies involving non-human teeth or permanent teeth were excluded, as the research specifically targeted primary teeth. Research using stainless steel instruments for root canal preparation or evaluating dentinal cracks after root canal filling was also excluded. Furthermore, studies addressing retreatment scenarios were omitted to ensure that the included research provided detailed and focused data for analysis.

Data management and extraction

During the first step of screening, two reviewers independently read the titles and abstracts of these 5145 records to determine their relevance based on established inclusion and exclusion criteria. Studies were included if they involved primary teeth, utilised NiTi files as the intervention, compared dentinal crack propagation, and reported on the outcome of dentinal microcracks. Exclusion criteria encompassed studies on permanent teeth, those not involving a comparison group, and those lacking relevant outcome measures. This screening process resulted in the exclusion of 5130 studies, leaving 15 full-text articles for further assessment.

**Figure 1:**
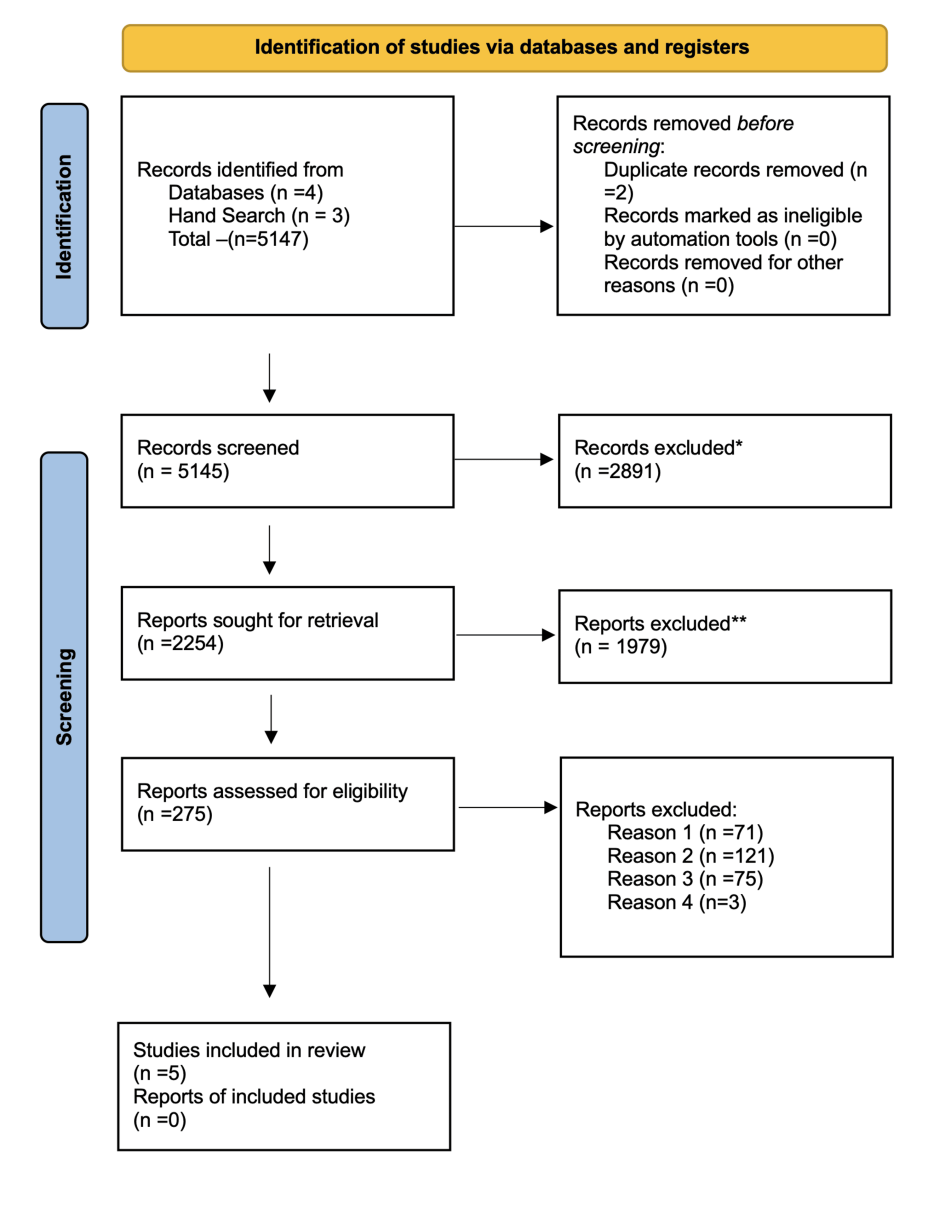
PRISMA flowchart PRISMA: Preferred Reporting Items for Systematic Reviews and Meta-Analyses *Articles from other fields except the medical field; ** articles from the medical field except dental; Reason 1: permanent teeth; Reason 2: other languages; Reason 3: irrelevant methodology; Reason 4: insufficient data

The full-text publications were then evaluated independently by the same two reviewers. During this step, studies were thoroughly examined to ensure that they met all inclusion criteria and to obtain detailed information on their methodology, interventions, comparisons, and results. Any disagreements among the reviewers were handled by discussion and consensus, with a third reviewer consulted if needed. This rigorous examination resulted in the elimination of 10 studies, largely due to inadequate data reporting, poor study design, or inability to meet the inclusion criteria. Finally, five papers were found appropriate for inclusion in the qualitative synthesis (Figure 1).

Data will be extracted using a standardised data extraction form, which will include the following information:

The study's characteristics, including the author and year of publication, were carefully documented, along with the study design. A comparison was made regarding the incidence of crack propagation. The outcome measures focused on micro-crack propagation, which was evaluated using various advanced techniques such as micro-CT and scanning electron microscopy, among others. A summary of the findings is shown in Table 1.

**Table 1:** Summary of the articles reviewed

Authors /Year	Study Design	Sample Size and Tooth Used	Interventions	Comparators	Method of Examination	Key Findings
Topkara et al. [7]	Experimental, comparative in vitro study	80 primary mandibular molars	Reciproc, Waveone Gold and Protaper next	K-files	Stereomicroscope	The Reciproc system showed the maximum incidence of defects compared to other groups
Patil et al. 2023 [8]	Experimental, comparative in vitro study	120 primary mandibular molars	Protaper gold, kedo-s rotary files	K-files	Stereomicroscope	More cracks were seen on the upper surface than on the lower surface in both groups.
Panda et al. 2021 [9]	Experimental, comparative in vitro study	60 primary molars	Protaper Universal, SAFs	K-files	Stereomicroscope	The ProTaper group showed the maximum incidence of defects compared to other groups.
Yuksel et al. 2021 [10]	Experimental, comparative in vitro study	30 primary molars	One shape, XP-endoshaper, and WaveOne Gold	_	Micro-CT	XP-endo Shaper exhibited the highest incidence of defects
Bhagyashree et al. 2021 [11]	Experimental, comparative in vitro study	150 primary anteriors	Pedoflex, Prime Pedo™, Kedo-S2	K-files	Stereomicroscope	Maximum crack formations in the middle third by Prime Pedo™

Risk of bias

**Figure 2:**
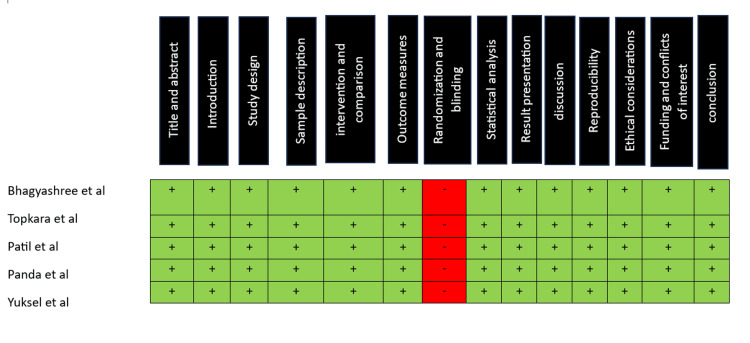
Risk of bias (+) represents low risk of bias, and (-) represents high risk of bias. Overall risk of bias evaluation was according to CRIS guidelines Articles by Topkara et al., Patil et al., Panda et al., Yuksel et al., and Bhagyashree et al. have a low risk of bias [7-11].

The risk of bias was evaluated based on the CRIS guidelines for in vitro studies. Based on the assessment, the articles have a low risk of bias (Figure 2).

Discussion

This systematic review comprehensively examines the effects of various NiTi rotary file systems on dentinal crack formation during root canal preparation in primary teeth. Preserving primary teeth is essential to maintaining both function and aesthetics until their natural exfoliation. The unique anatomy of primary teeth, with their thinner dentinal walls and complex root morphology, necessitates careful selection of instrumentation to ensure successful endodontic outcomes. The studies carried out by Bhagyashree et al., Topkara et al., Patil et al., Panda et al. and Yuksel et al. showed that the choice of rotary file system was shown to significantly influence the extent of dentinal damage, which in turn affects the long-term structural integrity of the tooth [7-11].

Several NiTi rotary file systems were examined, including ProTaper, WaveOne Gold, Reciproc, Kedo-S, One Shape, XP-Endo Shaper, and the Self-Adjusting File (SAF). Each system's design-especially the taper, cross-section, and motion characteristics-play a critical role in the degree of stress exerted on the dentinal walls, resulting in varying levels of dentinal crack formation.

In a notable finding, Topkara et al. [7] reported that the Reciproc system induced more cracks, particularly in the apical region, due to its S-shaped cross-section and larger taper. The greater taper likely contributes to increased stress on the canal walls, which may cause more significant dentinal damage. On the other hand, the WaveOne Gold system, with its parallelogram-shaped cross-section and reduced taper, produced fewer cracks. Its off-centred design minimises contact with the canal walls, reducing mechanical stress on the dentin.

Patil et al. [8] compared ProTaper Gold with Kedo-S, a system specifically designed for paediatric patients. Although ProTaper Gold demonstrated effective cleaning and shaping capabilities, it resulted in more dentinal cracks than Kedo-S. The Kedo-S file’s smaller taper and shorter working length made it more suitable for the narrow, curved canals of primary teeth, reducing the likelihood of crack formation.

In another study, Panda et al. [9] demonstrated that ProTaper Universal rotary files led to significantly more microcracks compared to both hand files and the SAF system. The SAF system, which operates with a non-rotational, scraping motion, showed superior performance in minimising dentinal damage. Unlike ProTaper’s continuous rotational force, the SAF system adapts to the canal’s natural shape, reducing the risk of overpreparation and minimising stress on the dentin.

Yüksel et al. [10] introduced a detailed micro-CT evaluation of primary molars prepared with the One Shape, XP-Endo Shaper, and WaveOne Gold systems. The study found that WaveOne Gold caused the highest reduction in dentin thickness, particularly in the danger zone of the mesial root canals. This is mainly because the mesiodistal dimension is less compared to that of the linguobuccal diameter. The XP-Endo Shaper generated the most microcracks, while One Shape caused significantly fewer cracks and less dentin removal. These findings underscore the potential risks of excessive dentin removal during root canal preparation, especially in vulnerable areas like the danger zone, which are prone to fractures.

Bhagyashree et al. [11] corroborate these observations by showing that hand files, although slower, caused fewer dentinal cracks than rotary systems. Manual instrumentation, with its controlled, less aggressive action, may be preferable for fragile primary teeth. However, the longer procedure time associated with hand instrumentation presents challenges, especially in paediatric patients, where cooperation may be limited.

Overall, across all studies in our review, a consistent pattern emerges: file systems with larger tapers and more aggressive cutting actions, such as ProTaper, Reciproc, and XP-Endo Shaper, tend to induce more dentinal cracks. Meanwhile, systems designed for greater flexibility, such as Kedo-S, One Shape, and SAF, demonstrate better outcomes for preserving dentinal integrity. These findings underscore the importance of selecting the appropriate file system based on the specific anatomical needs of primary teeth.

In this systematic review, 793 primary teeth samples were analysed, with mandibular molars being the most commonly studied teeth. Three studies recorded samples with root curvature, while others primarily focused on teeth with straight roots. Root curvature specifics were clearly defined only by Yüksel et al., while other studies provided limited details on this aspect. In the articles we are reviewing, the ProTaper system was the most commonly used NiTi file system, followed by WaveOne Gold. Among M-wire systems, WaveOne Gold was the most frequently employed in the five studies, with ProTaper Next and Reciproc following closely behind. The SAF system and Kedo-S, both designed to minimise dentinal damage, were used less frequently across the studies reviewed [8-10].

The presence of dentinal microcracks was evaluated at various cross-sections using micro-CT and SEM. It was found that there was a higher number of defects with the use of the ProTaper Universal system. These defects were seen at the 3 mm section from the root apex. Similarly, WaveOne Gold showed a higher incidence of microcracks among the M-wire systems. Four studies using ProTaper Next reported an increase in the number of microcracks at multiple cross-sections, particularly at 6 mm and 9 mm levels [8-10]. The SAF system demonstrated fewer microcracks across all studies, consistent with findings from Yüksel et al., while Kedo-S also showed promising results in reducing dentinal defects [8]. It should be noted that SAF and Kedo-S were among the least frequently used systems in the studies reviewed. Across all studies, the highest occurrence of microcracks was noted at the 3 mm section from the root apex, with a marked decrease in cracks as the distance from the apex increased. This might be due to the anatomy of the root structure, i.e., the anatomical root starts to narrow down from the cervical region till it reaches the apex.

The clinical implications of these findings are significant, particularly for pediatric endodontics, where maintaining the long-term retention of primary teeth is crucial. Dentinal cracks formed during root canal preparation can act as precursors to vertical root fractures that compromise the treatment's success and may ultimately lead to tooth loss. This is especially critical in primary teeth, where maintaining structural integrity is necessary until the permanent successors erupt. Additionally, vertical root fractures in primary teeth can cause pain, infection, and premature extraction, leading to space loss, malocclusion, and aesthetic concerns for the child.

Patil et al. [8] underscore the importance of using file systems designed specifically for paediatric teeth, like Kedo-S, to minimise dentinal damage and reduce the risk of over-preparation. The smaller taper and shorter working length of Kedo-S ensure that it is more suited for primary teeth, where the thinner dentinal walls make them more vulnerable to cracks. Panda et al. [9] and Yüksel et al. [10] support these conclusions, highlighting that SAF and One Shape systems perform better in preserving dentinal structure than more aggressive file systems like ProTaper or XP-Endo Shaper. SAF’s non-rotational motion and constant irrigation reduce the risk of vertical fractures, making it an excellent option for cases where dentinal preservation is critical.

Bhagyashree et al. [11] add that hand instrumentation, though time-consuming, results in fewer cracks compared to rotary file systems. This suggests that less aggressive mechanical forces during preparation may reduce stress on the dentinal walls, particularly in younger patients with primary teeth. However, manual instrumentation also comes with challenges, especially for pediatric patients, due to longer procedure times and the need for patient cooperation.

Meanwhile, Topkara et al. [7] and Patil et al. [8] emphasise that rotary systems like WaveOne Gold, which demonstrate reduced crack formation while maintaining high efficiency, may offer a practical compromise between safety and speed. Paediatric patients often have limited attention spans, making timely treatment essential. Therefore, clinicians must carefully balance the benefits of minimising dentinal cracks with the need for efficient and timely treatment.

Although the findings of this systematic review provide valuable insights, several limitations must be acknowledged. The studies included were conducted in vitro, where controlled conditions may not fully replicate the complexities of clinical practice. Factors such as patient movement, anatomical variability, and pre-existing conditions like root resorption can influence treatment outcomes in vivo. Additionally, variations in the methodologies used to evaluate dentinal cracks, such as magnification levels and imaging techniques, introduce variability in the results, affecting comparability across studies.

Standardising the methodologies for assessing dentinal cracks across studies would improve consistency and allow for more robust comparisons. Future research should focus on clinical trials to validate these in vitro results and explore how different rotary systems perform in real-world paediatric dental settings. Further studies should also consider the anatomical variations specific to primary teeth. The future of paediatric endodontics lies in refining file systems to reduce the risk of dentinal damage while improving treatment outcomes. Systems like Kedo-S and One Shape have already demonstrated that designing files specifically for the anatomy of primary teeth-considering shorter lengths, smaller tapers, and enhanced flexibility-can lead to better clinical results. Studies conducted by Panja et al. on improving files using GO coating can also lead to better results [12,13]. However, further research needs to be carried out to explore other properties of these paediatric rotary files. This will enable the research efforts to focus on improving the safety, effectiveness, and reliability of endodontic treatments for paediatric patients.

## Conclusions

NiTi rotary file systems, particularly those designed with paediatric considerations like Kedo-S, One Shape, and SAF, have shown a significant promise in reducing dentinal crack formation during root canal preparation in primary teeth. Our review found that paediatric rotary files like Wave One, Kedo S, SAF, and One Shape experience less dentinal cracking when used in primary root canals. This is because of their flexibility and small size. These systems offer a balanced approach, combining efficient canal shaping with the preservation of dentinal integrity, which is crucial for maintaining primary teeth until their natural exfoliation. The findings of this review highlight the importance of selecting the appropriate instrumentation based on the anatomical challenges of primary teeth. Considering the use of these files in our practices to minimise crack propagation and enhance treatment success rates. This will benefit both our patients and our overall practice efficiency. 

## References

[1] Peters OA, Barbakow F, Peters CI (2004). An analysis of endodontic treatment with three nickel-titanium rotary root canal preparation techniques. Int Endod J.

[2] Shen Y, Zhou HM, Zheng YF, Peng B, Haapasalo M (2013). Current challenges and concepts of the thermomechanical treatment of nickel-titanium instruments. J Endod.

[3] Gambarini G, Grande NM, Plotino G, Somma F, Garala M, De Luca M, Testarelli L (2008). Fatigue resistance of engine-driven rotary nickel-titanium instruments produced by new manufacturing methods. J Endod.

[4] Rodd HD, Waterhouse PJ, Fuks AB, Fayle SA, Moffat MA (2006). Pulp therapy for primary molars. Int J Paediatr Dent.

[5] Erkan E, Olcay K, Eyüboğlu TF, Gündoğar M (2019). Incidence of dentinal crack formation during root canal preparation with two NiTi instruments activated by adaptive motion and continuous rotation: an in vitro study. Eur Res J.

[6] Saberi EA, Mollashahi NF, Ahmadi M (2019). Comparative evaluation of dentinal microcracks in root canals prepared by Neoniti, Reciproc, and ProTaper instruments. Zahedan J Res Med Sci.

[7] Topkara C, Özyürek T, Demiryürek EO, Bursalı T, Özler M (2017). Attitudes, materials, and methods preferred in root canal treatment in Turkey: a survey. Turkish Endod J.

[8] Patil MB, Mandroli PS, Jalannavar P, Patil BB (2023). Dentinal microcracks after root canal preparation in primary root: an in vitro evaluation of ProTaper Gold and Kedo-S rotary file systems. Int J Clin Pediatr Dent.

[9] Panda A, Shah K, Budakoti V, Dere K, Virda M, Jani J (2021). Evaluation of microcrack formation during root canal preparation using hand, rotary files and self-adjusting file in primary teeth: an in vitro study. J Dent Res Dent Clin Dent Prospects.

[10] Yüksel BN, Öncü A, Çelİkten B, Bİlecenoğlu B, Orhan AI, Orhan K (2022). Micro-CT evaluation of 'danger zone' and microcrack formation in mesial root canals of primary teeth with single-file rotary and reciprocating systems. Int J Paediatr Dent.

[11] Bhagyashree B, Rao D, Panwar S, Kothari N, Gupta S (2022). An in vitro comparative evaluation of dentinal crack formation caused by three different nickel-titanium rotary file systems in primary anterior teeth. J Indian Soc Pedod Prev Dent.

[12] Panja K, A VS, N V, Ramar K (2024). Surface coating of nickel-titanium (Ni-Ti) pediatric rotary file using graphene oxide: a scanning electron microscopy analysis. Cureus.

[13] Panja K, Victor A, Vivek N (2024). Elemental analysis of a nickel-titanium (Ni-Ti) pediatric rotary file coated with graphene oxide: an energy dispersive X-ray analysis. Cureus.

